# Supplementary material for: Genetic and Molecular Characterization of the Immortalized Murine Hepatic Stellate Cell Line GRX
Source: Cells. 2022 Apr 30;11(9):1504. doi: 10.3390/cells11091504 (PMC9102025; doi:10.3390/cells11091504)
Supplement: Supplementary file 1 [file cells-11-01504-s001.zip › Supplementary Figures.pdf]

Supplementary Figures

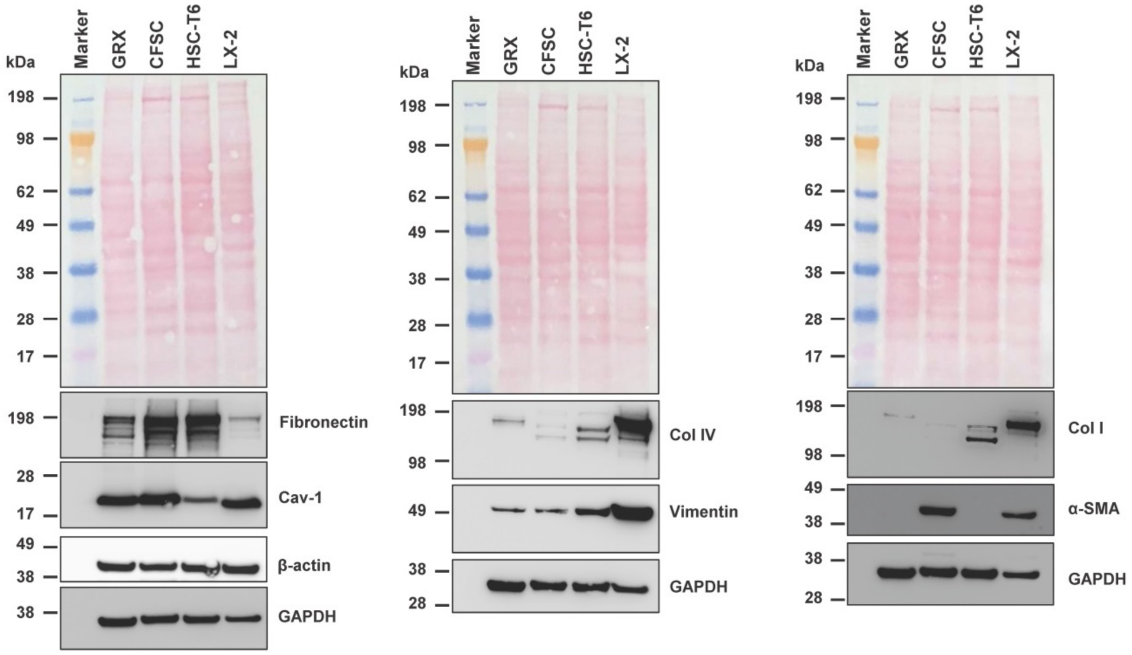

Figure S1. Comparison of marker protein expression in different HSC lines by Western blot analysis. Cell extracts were prepared from murine (GRX), rat (CFSC, HSC-T6) and human (LX-2) HSC lines and analyzed for expression of α-smooth muscle actin (α-SMA), caveolin-1 (Cav-1), collagen type I (Col I), collagen type IV (Col IV), fibronectin and vimentin. The expression of β-actin or GAPDH and the Ponceau S stain were included to demonstrate equal protein loading. Please note that the expression of α-SMA in GRX cells in the depicted blot is hardly detectable since α-SMA is strongly expressed by CFSC and LX-2 cells and that the obtained signals are not directly comparable because of the different specificities of antibodies used.

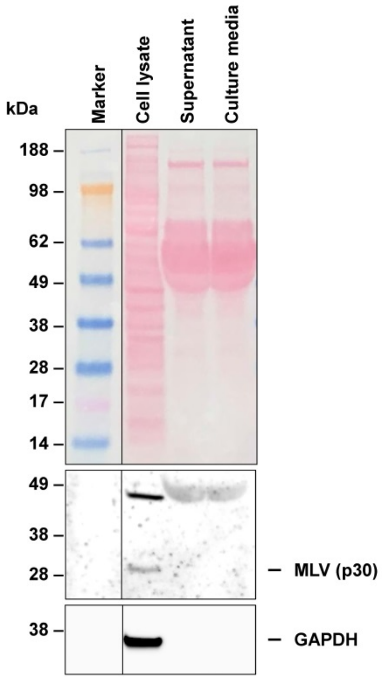

Figure S2. Western blot analysis for p30 gag protein of murine leukemia virus. Protein extracts and supernatant of cultures GRX cells were probed by Western blot with an antibody directed against MLV (p30). The protein was re-probed with a GAPDH specific antibody. Ponceau S stain is shown to demonstrate integrity of proteins. Please note that in supernatants no p30 is detectable suggesting that most viral particles are attached to the cells.

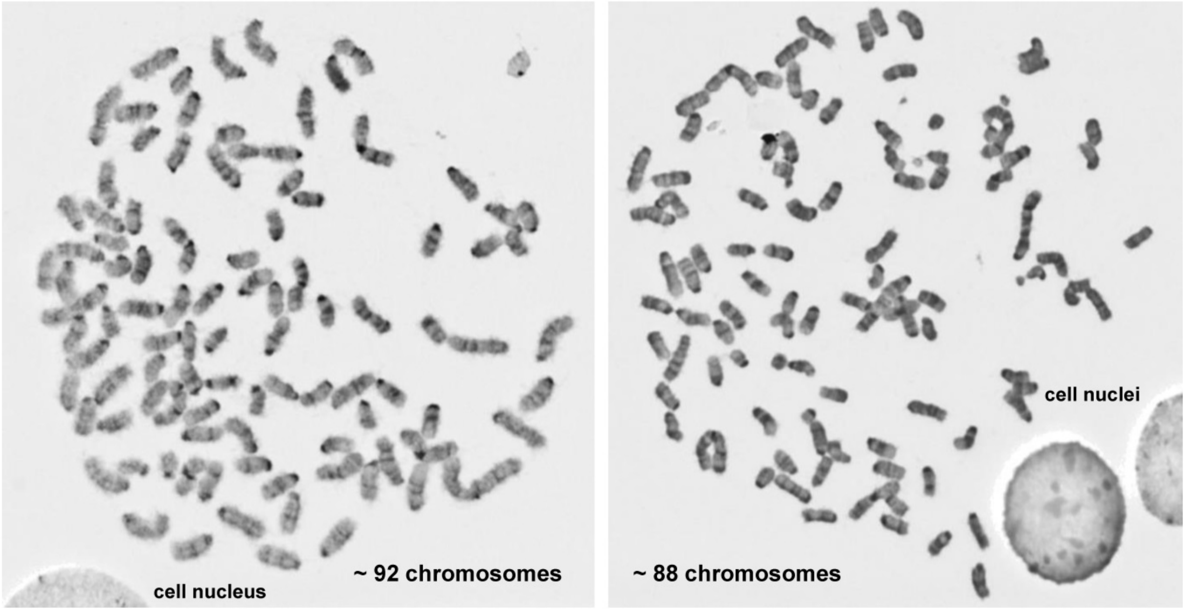

Figure S3. Occurrence of polyploidy in GRX cells. Representative somatic metaphases showing polyploidy that frequently occur in GRX cells. In the depicted examples, the number of chromosomes observed was almost a hundred.

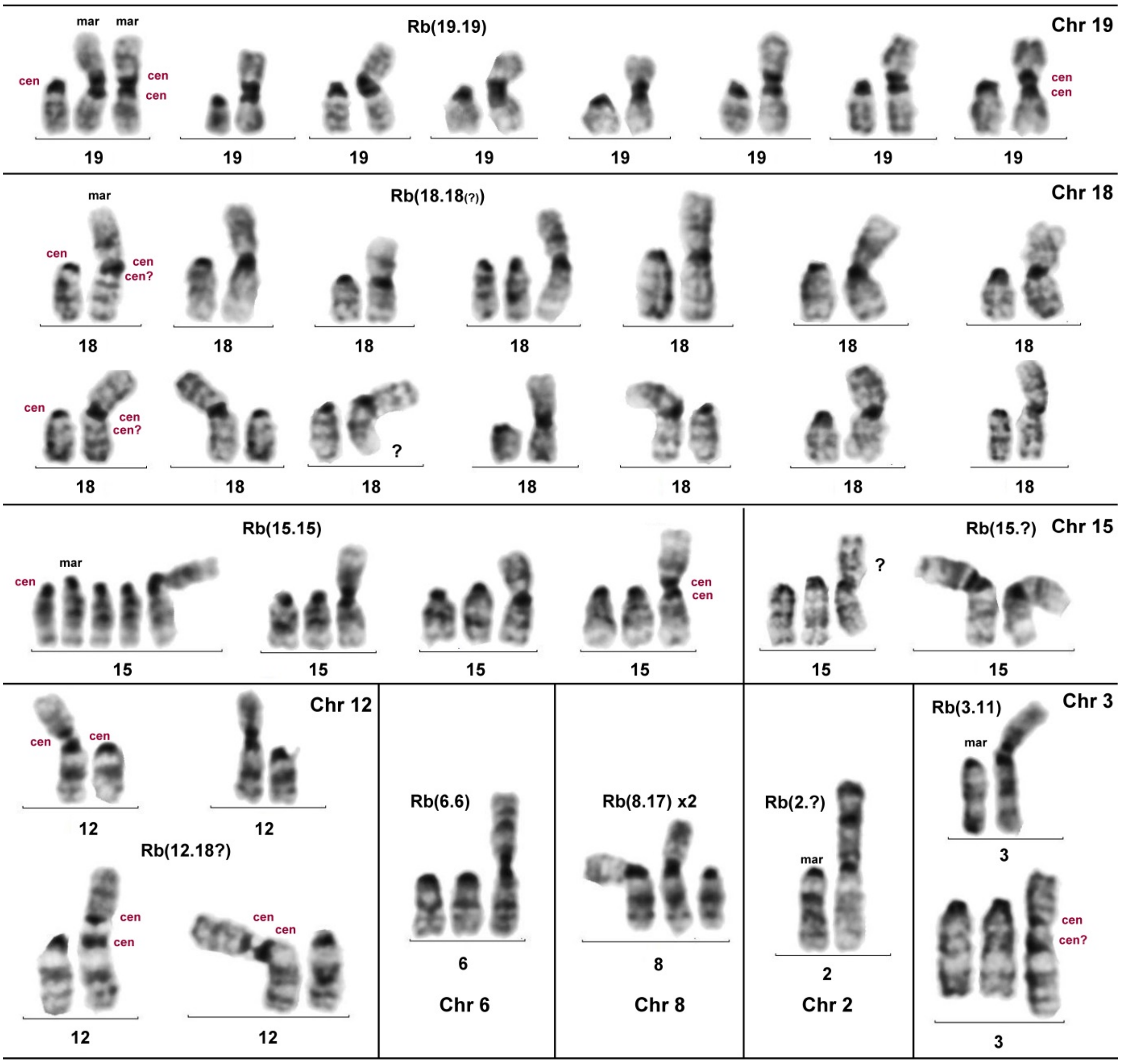

Figure S4. Robertsonian translocations in GRX cells. Karyotype analysis revealed that Robertsonian (Rb) translocations occurred in high frequency in GRX cells. These chromosomal abnormalities affected chromosomes with terminal or near-terminal centromeres, namely Chr 19, Chr 18, Chr 15, Chr 12, Chr 6, Chr 8, Chr 2, and Chr 3.

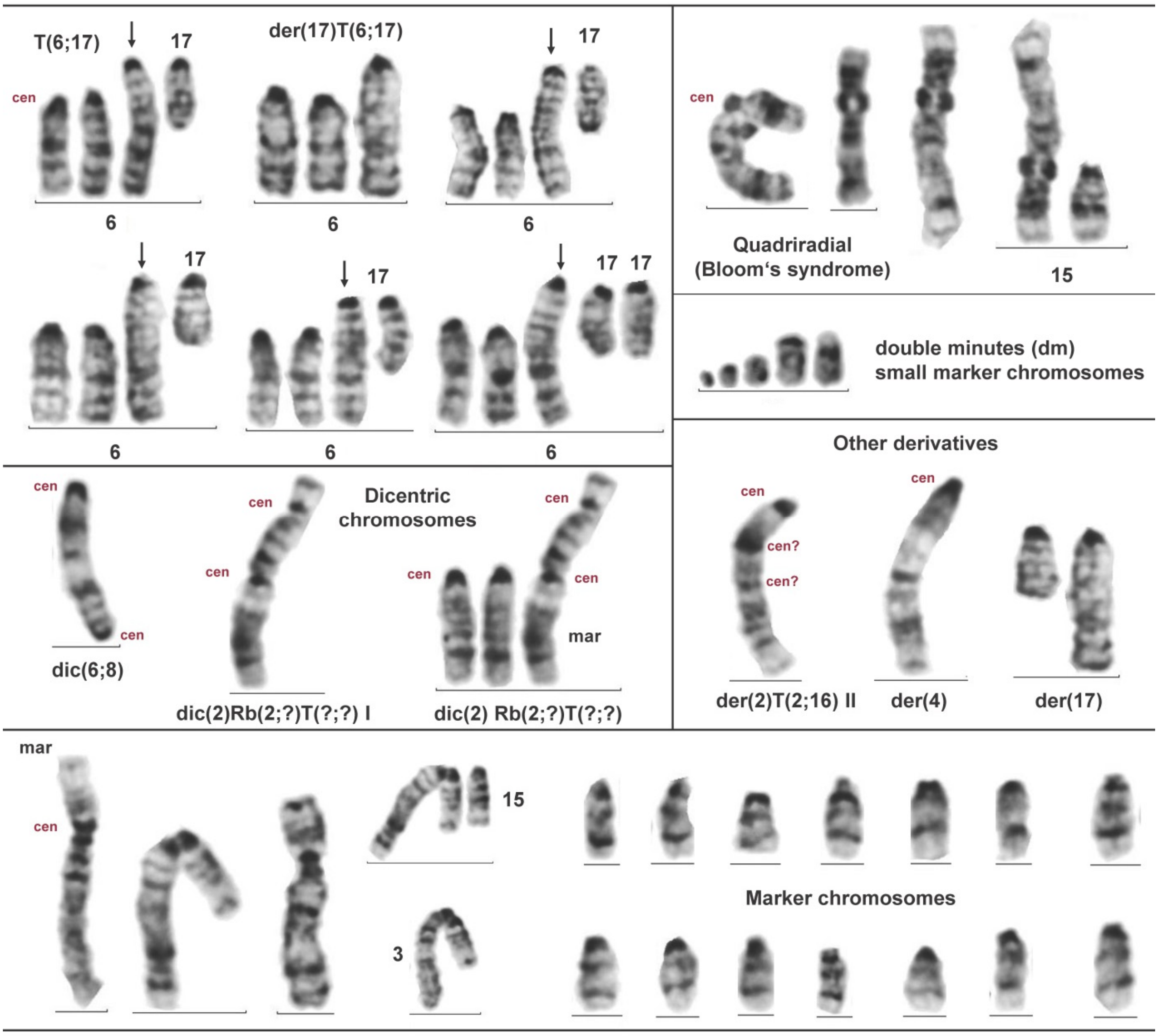

Figure S5. Additional chromosomal arrangements in GRX cells. Besides Robertsonian translocations, GRX cells contain many other chromosomal abnormalities. Frequently observed were more defined translocations between Chr 6 and Chr 17, dicentric chromosomes, small marker chromosomes, multicentromeric chromosomes, double minutes, and quadriradial chromosomes composed of four arms.
