# Supplementary material for: Genetic and Molecular Characterization of the Immortalized Murine Hepatic Stellate Cell Line GRX
Source: Cells. 2022 Apr 30;11(9):1504. doi: 10.3390/cells11091504 (PMC9102025; doi:10.3390/cells11091504)
Supplement: Supplementary file 1 [file cells-11-01504-s001.zip › Table S1.pdf]

Table S1. Publications using or mentioning GRX cells

S/N	Year of publication	Reference	Type of paper	Origin of publication
1	1985	Borojevic R, Monteiro AN, Vinhas SA, Domont GB, Mourão PA, Emonard H, Grimaldi G Jr, Grimaud JA. Establishment of a continuous cell line from fibrotic schistosomal granulomas in mice livers. In Vitro Cell Dev Biol. 1985;21(7):382-390. doi: 10.1007/BF02623469	Original	Brazil
2	1989	Margis R, Borojevic R. Retinoid-mediated induction of the fat-storing phenotype in a liver connective tissue cell line (GRX). Biochim Biophys Acta 1989;1011(1):1-5. doi: 10.1016/0167-4889(89)90069-4	Original	Brazil
3	1990	Borojevic R, Guaragna RM, Margis R, Dutra HS. In vitro induction of the fat-storing phenotype in a liver connective tissue cell line-GRX. In Vitro Cell Dev Biol. 1990;26(4):361-368. doi: 10.1007/BF02623827	Original	Brazil
4	1990	Silva LC, Borojevic R, Mourão PA. Identification of cells responsible for synthesis of sulphated glycosaminoglycans in schistosome-induced hepatic granulomas. Int J Exp Pathol. 1990;71(6):845-856. PMID: 2126187	Original	Brazil
5	1991	Guaragna RM, Trugo L, Borojevic R. Neutral lipid synthesis and accumulation during in vitro induction of the lipocyte phenotype in hepatic connective tissue cells. Biochim Biophys Acta 1991;1085(1):29-34. doi: 10.1016/0005-2760(91)90228-a	Original	Brazil
6	1992	Margis R, Pinheiro-Margis M, da Silva LC, Borojevic R. Effects of retinol on proliferation, cell adherence and extracellular matrix synthesis in a liver myofibroblast or lipocyte cell line (GRX). Int J Exp Pathol. 1992;73(2):125-135. PMID: 1571273	Original	Brazil
7	1992	Pinheiro-Margis M, Margis R, Borojevic R. Collagen synthesis in an established liver connective tissue cell line (GRX) during induction of the fat-storing phenotype. Exp Mol Pathol. 1992;56(2):108-118. doi: 10.1016/0014-4800(92)90028-a	Original	Brazil
8	1992	Silva LC, Borojevic R, Mourão PA. Proteoglycans synthesized by the hepatic granulomas isolated from schistosome-infected mice and by the granuloma-derived connective tissue cell lines. Biochim Biophys Acta 1992;1139(1-2):96-104. doi: 10.1016/0925-4439(92)90088-5	Original	Brazil
9	1992	Guaragna RM, Trugo L, Borojevic R. Phospholipid modifications during conversion of hepatic myofibroblasts into lipocytes (Ito-cells). Biochim Biophys Acta 1992;1128(2-3):237-243. doi: 10.1016/0005-2760(92)90313-k	Original	Brazil
10	1995	Monteiro AN, Geremias AT, Borojevic R. Clonal heterogeneity in murine liver myofibroblasts. Braz J Med Biol Res. 1995;28(4):457-466. PMID: 8520543	Original	Brazil
11	1998	Vicente CP, Fortuna VA, Margis R, Trugo L, Borojevic R. Retinol uptake and metabolism, and cellular retinol binding protein expression in an in vitro model of hepatic stellate cells. Mol Cell Biochem. 1998;187(1-2):11-21. doi: 10.1023/a:1006886308490	Original	Brazil
12	1999	Kato M, Iwamoto H, Higashi N, Sugimoto R, Uchimura K, Tada S, Sakai H, Nakamuta M, Nawata H. Role of Rho small GTP binding protein in the regulation of actin cytoskeleton in hepatic stellate cells. J Hepatol. 1999;31(1):91-99. doi: 10.1016/s0168-8278(99)80168-8	Original	Japan
13	2001	Geerts A. History, heterogeneity, developmental biology, and functions of quiescent hepatic stellate cells. Semin Liver	Review	UK

		Dis. 2001;21(3):311-335. doi: 10.1055/s-2001-17550		
14	2001	Guma FCR, Mello TG, Mermelstein CS, Fortuna VA, Wofchuk ST, Gottfried C, Guaragna RM, Costa ML, Borojevic R. Intermediate filaments modulation in an in vitro model of the hepatic stellate cell activation or conversion into the lipocyte phenotype. Biochem Cell Biol. 2001;79(4):409-417. PMID: 11527210	Original	Brazil
15	2001	Mermelstein CS, Guma FC, Mello TG, Fortuna VA, Guaragna RM, Costa ML, Borojevic R. Induction of the lipocyte phenotype in murine hepatic stellate cells: reorganisation of the actin cytoskeleton. Cell Tissue Res. 2001;306(1):75-83. doi: 10.1007/s004410100428	Original	Brazil
16	2001	Fortuna VA, Trugo LC, Borojevic R. Acyl-CoA: retinol acyltransferase (ARAT) and lecithin:retinol acyltransferase (LRAT) activation during the lipocyte phenotype induction in hepatic stellate cells. J Nutr Biochem. 2001;12(11):610-621. doi: 10.1016/s0955-2863(01)00179-6	Original	Brazil
17	2003	Andrade CM, Trindade VM, Cardoso CC, Ziulkoski AL, Trugo LC, Guaragna RM, Borojevic R, Guma FC. Changes of sphingolipid species in the phenotype conversion from myofibroblasts to lipocytes in hepatic stellate cells. J Cell Biochem. 2003;88(3):533-544. doi: 10.1002/jcb.1037	Original	Brazil
18	2003	da Silva FM, Guimarães EL, Grivicich I, Trindade VM, Guaragna RM, Borojevic R, Guma FC. Hepatic stellate cell activation in vitro: cell cycle arrest at G2/M and modification of cell motility. J Cell Biochem. 2003;90(2):387-396. doi: 10.1002/jcb.10642	Original	Brazil
19	2003	Fortuna VA, Martucci RB, Trugo LC, Borojevic R. Hepatic stellate cells uptake of retinol associated with retinol-binding protein or with bovine serum albumin. J Cell Biochem. 2003;90(4):792-805. doi: 10.1002/jcb.10703	Original	Brazil
20	2004	Martucci RB, Ziulkoski AL, Fortuna VA, Guaragna RM, Guma FC, Trugo LC, Borojevic R. Beta-carotene storage, conversion to retinoic acid, and induction of the lipocyte phenotype in hepatic stellate cells. J Cell Biochem. 2004;92(2):414-423. doi: 10.1002/jcb.20073	Original	Brazil
21	2007	Van de Bovenkamp M, Groothuis GM, Meijer DK, Olinga P. Liver fibrosis in vitro: cell culture models and precision-cut liver slices. Toxicol In Vitro. 2007;21(4):545-557. doi: 10.1016/j.tiv.2006.12.009	Review	The Netherlands
22	2007	Herrmann J, Gressner AM, Weiskirchen R. Immortal hepatic stellate cell lines: useful tools to study hepatic stellate cell biology and function? J Cell Mol Med. 2007;11(4):704-722. doi: 10.1111/j.1582-4934.2007.00060.x	Review	Germany
23	2007	Guimarães EL, Franceschi MF, Andrade CM, Guaragna RM, Borojevic R, Margis R, Bernard EA, Guma FC. Hepatic stellate cell line modulates lipogenic transcription factors. Liver Int. 2007;27(9):1255-1264. doi: 10.1111/j.1478-3231.2007.01578.x	Original	Brazil
24	2008	Friedman SL. Hepatic stellate cells: protean, multifunctional, and enigmatic cells of the liver. Physiol Rev. 2008;88(1):125-172. doi: 10.1152/physrev.00013.20	Review	USA
25	2008	Andrade CM, Roesch GC, Wink MR, Guimarães EL, Souza LF, Jardim FR, Guaragna RM, Bernard EA, Margis R, Borojevic R, Battastini AM, Guma FC. Activity and expression of ecto-5'-nucleotidase/CD73 are increased during phenotype conversion of a hepatic stellate cell line. Life Sci. 2008;82(1-2):21-29. doi: 10.1016/j.lfs.2007.10.003	Original	Brazil
26	2008	Souza IC, Martins LA, Coelho BP, Grivicich I, Guaragna RM, Gottfried C, Borojevic R, Guma FC. Resveratrol inhibits cell growth by inducing cell cycle arrest in activated hepatic stellate cells. Mol Cell Biochem. 2008;315(1-2):1-7. doi:	Original	Brazil

		10.1007/s11010-008-9781-x		
27	2009	Karlmark KR, Weiskirchen R, Zimmermann HW, Gassler N, Ginhoux F, Weber C, Merad M, Luedde T, Trautwein C, Tacke F. Hepatic recruitment of the inflammatory $Gr1^+$ monocyte subset upon liver injury promotes hepatic fibrosis. Hepatology 2009;50(1):261-274. doi: 10.1002/hep.22950	Original	Germany
28	2009	Andrade CM, Wink MR, Margis R, Borojevic R, Battastini AM, Guma FC. Activity and expression of ecto-nucleotide pyrophosphate/phosphodiesterases in a hepatic stellate cell line. Mol Cell Biochem. 2009;325(1-2):179-185. doi: 10.1007/s11010-009-0032-6	Original	Brazil
29	2009	Teodoro AJ, Perrone D, Martucci RB, Borojevic R. Lycopene isomerisation and storage in an in vitro model of murine hepatic stellate cells. Eur J Nutr. 2009;48(5):261-268. doi: 10.1007/s00394-009-0001-6	Original	Brazil
30	2010	Berres ML, Koenen RR, Rueland A, Zaldivar MM, Heinrichs D, Sahin H, Schmitz P, Streetz KL, Berg T, Gassler N, Weiskirchen R, Proudfoot A, Weber C, Trautwein C, Wasmuth HE. Antagonism of the chemokine Ccl5 ameliorates experimental liver fibrosis in mice. J Clin Invest. 2010;120(11):4129-4140. doi: 10.1172/JCI41732	Original	Germany
31	2010	Andrade CM, Wink MR, Margis R, Borojevic R, Battastini AM, Guma FC. Changes in E-NTPDase 3 expression and extracellular nucleotide hydrolysis during the myofibroblast/lipocyte differentiation. Mol Cell Biochem. 2010;339(1-2):79-87. doi: 10.1007/s11010-009-0371-3	Original	Brazil
32	2011	Gamarra ML, Albuquerque MCM, Teodore AJ, Martucci RB, Borjevic R, Camara FP, Romanos MTV, Santos N. Susceptibility of a continuous murine cell line (GRX) to viral infection. Rev Pan-Amaz Saude 2011;2:65-69. doi: 10.5123/S2176-62232011000200009	Original	Brazil
33	2011	Baldo G, Kretzmann NA, Tieppo J, Filho GP, Cruz CU, Meurer L, da Silveira TR, dos Santos JL, Marroni NP, Giugliani R, Matte U. Bone marrow cells reduce collagen deposition in the rat model of common bile duct ligation. J Cell Sci Ther 2011;2:112. doi:10.4172/2157-7013.1000112	Original	Brazil
34	2011	Scholten D, Al-samman M, Sahin H, Trautwein C, Wasmuth HE. CXCR3 ligands induce expression of CXCL1 (KC/murine IL8 homolog) in mouse hepatic stellate cells. J Cell Sci Ther 2011;S5. doi: 10.4172/2157-7013.S5-00	Original	Germany
35	2011	Stefano JT, Cogliati B, Santos F, Lima VM, Mazo DC, Matte U, Alvares-da-Silva MR, Silveira TR, Carrilho FJ, Oliveira CP. S-Nitroso-N-acetylcysteine induces de-differentiation of activated hepatic stellate cells and promotes antifibrotic effects in vitro. Nitric Oxide 2011;25(3):360-365. doi: 10.1016/j.niox.2011.07.00	Original	Brazil
36	2012	O Al-Harbi N, Bahashwan SA, Aboonq MS, Ramadan MA, Bahashwan AA. Reversal of liver fibrosis in chronic murine Schistosomiasis mansoni by Safironil/Praziquantel. Trop J Pharm Res. 2012; 11: 537-543. doi: 10.4314/tjpr.v11i4.3	Original	Saudi Arabia
37	2012	Nellen A, Heinrichs D, Berres ML, Sahin H, Schmitz P, Proudfoot AE, Trautwein C, Wasmuth HE. Interference with oligomerization and glycosaminoglycan binding of the chemokine CCL5 improves experimental liver injury. PLoS One. 2012;7(5):e36614. doi: 10.1371/journal.pone.0036614	Original	Germany
38	2012	de Moraes CMB, Melo DA, Santos RC, Bitencourt S, Mesquita FC, dos Santos de Oliveira F, Rodriguez-Carballo E, Bartrons R, Rosa JL, Ventura FP, Rodrigues de Oliveira J. Antiproliferative effect of catechin in GRX cells. Biochem Cell Biol. 2012;90(4):575-584. doi: 10.1139/o2012-010	Original	Brazil

39	2012	Bitencourt S, de Mesquita FC, Caberlon E, da Silva GV, Basso BS, Ferreira GA, de Oliveira JR. Capsaicin induces de-differentiation of activated hepatic stellate cell. Biochem Cell Biol. 2012;90(6):683-690. doi: 10.1139/o2012-026	Original	Brazil
40	2013	Meurer SK, Alsamman M, Sahin H, Wasmuth HE, Kisseleva T, Brenner DA, Trautwein C, Weiskirchen R, Scholten D. Overexpression of endoglin modulates TGF-β1-signalling pathways in a novel immortalized mouse hepatic stellate cell line. PLoS One. 2013;8(2):e56116. doi: 10.1371/journal.pone.0056116	Original	Germany
41	2013	Heinrichs D, Berres ML, Nellen A, Fischer P, Scholten D, Trautwein C, Wasmuth HE, Sahin H. The chemokine CCL3 promotes experimental liver fibrosis in mice. PLoS One. 2013;8(6):e66106. doi: 10.1371/journal.pone.0066106	Original	Germany
42	2013	Roderburg C, Luedde M, Vargas Cardenas D, Vucur M, Mollnow T, Zimmermann HW, Koch A, Hellerbrand C, Weiskirchen R, Frey N, Tacke F, Trautwein C, Luedde T. miR-133a mediates TGF-β-dependent derepression of collagen synthesis in hepatic stellate cells during liver fibrosis. J Hepatol. 2013;58(4):736-742. doi: 10.1016/j.jhep.2012.11.022	Original	Germany
43	2013	Uribe CC, Dos Santos de Oliveira F, Grossmann B, Kretzmann NA, Reverbel da Silveira T, Giugliani R, Matte U. Cytotoxic effect of amphotericin B in a myofibroblast cell line. Toxicol In Vitro. 2013;27(7):2105-2109. doi: 10.1016/j.tiv.2013.08.005	Original	Brazil
44	2013	de Mesquita FC, Bitencourt S, Caberlon E, da Silva GV, Basso BS, Schmid J, Ferreira GA, de Oliveira Fdos S, de Oliveira JR. Fructose-1,6-bisphosphate induces phenotypic reversion of activated hepatic stellate cell. Eur J Pharmacol. 2013;720(1-3):320-325. doi: 10.1016/j.ejphar.2013.09.067	Original	Brazil
45	2014	Hammerich L, Bangen JM, Govaere O, Zimmermann HW, Gassler N, Huss S, Liedtke C, Prinz I, Lira SA, Luedde T, Roskams T, Trautwein C, Heymann F, Tacke F. Chemokine receptor CCR6-dependent accumulation of γδ T cells in injured liver restricts hepatic inflammation and fibrosis. Hepatology 2014;59(2):630-642. doi: 10.1002/hep.26697	Original	Germany
46	2014	Denardin CC, Parisi MM, Martins LA, Terra SR, Borojevic R, Vizzotto M, Perry ML, Emanuelli T, Guma FT. Antiproliferative and cytotoxic effects of purple pitanga (*Eugenia uniflora L.*) extract on activated hepatic stellate cells. Cell Biochem Funct. 2014;32(1):16-23. doi: 10.1002/cbf.2965	Original	Brazil
47	2014	Martins LA, Coelho BP, Behr G, Pettenuzzo LF, Souza IC, Moreira JC, Borojevic R, Gottfried C, Guma FC. Resveratrol induces pro-oxidant effects and time-dependent resistance to cytotoxicity in activated hepatic stellate cells. Cell Biochem Biophys. 2014;68(2):247-257. doi: 10.1007/s12013-013-9703-8	Original	Brazil
48	2014	de Moraes CMB, Bitencourt S, de Mesquita FC, Mello D, de Oliveira LP, da Silva GV, Lorini V, Caberlon E, de Souza Basso B, Schmid J, Ferreira GA, de Oliveira JR. (+)-Catechin attenuates activation of hepatic stellate cells. Cell Biol Int. 2014;38(4):526-530. doi: 10.1002/cbin.10228	Original	Brazil
49	2015	Martins LAM, Vieira MQ, Ilha M, de Vasconcelos M, Biehl HB, Lima DB, Schein V, Barbé-Tuana F, Borojevic R, Guma FC. The interplay between apoptosis, mitophagy and mitochondrial biogenesis induced by resveratrol can determine activated hepatic stellate cells death or survival. Cell Biochem Biophys. 2015;71(2):657-672. doi: 10.1007/s12013-014-0245-5	Original	Brazil
50	2015	Borkham-Kamphorst E, Alexi P, Tihaa L, Haas U, Weiskirchen R. Platelet-derived growth factor-D modulates extracellular matrix homeostasis and remodeling through TIMP-1 induction and attenuation of MMP-2 and MMP-9	Original	Germany

		gelatinase activities. Biochem Biophys Res Commun. 2015;457(3):307-313. doi: 10.1016/j.bbrc.2014.12.106		
51	2015	de Souza IC, Martins LA, de Vasconcelos M, de Oliveira CM, Barbé-Tuana F, Andrade CB, Pettenuzzo LF, Borojevic R, Margis R, Guaragna R, Guma FC. Resveratrol regulates the quiescence-like induction of activated stellate cells by modulating the PPARγ/SIRT1 ratio. J Cell Biochem. 2015;116(10):2304-2312. doi: 10.1002/jcb.2518	Original	Brazil
52	2016	Coombes JD, Choi SS, Swiderska-Syn M, Manka P, Reid DT, Palma E, Briones-Orta MA, Xie G, Younis R, Kitamura N, Della Peruta M, Bitencourt S, Dollé L, Oo YH, Mi Z, Kuo PC, Williams R, Chokshi S, Canbay A, Claridge LC, Eksteen B, Diehl AM, Syn WK. Osteopontin is a proximal effector of leptin-mediated non-alcoholic steatohepatitis (NASH) fibrosis. Biochim Biophys Acta 2016;1862(1):135-144. doi: 10.1016/j.bbadis.2015.10.028	Original	UK
53	2016	Yanguas SC, Cogliati B, Willebrords J, Maes M, Colle I, van den Bossche B, de Oliveira CPMS, Andraus W, Alves VAF, Leclercq I, Vinken M. Experimental models of liver fibrosis. Arch Toxicol. 2016;90(5):1025-1048. doi: 10.1007/s00204-015-1543-4	Review	Belgium
54	2017	Senoo H, Mezaki Y, Fujiwara M. The stellate cell system (vitamin A-storing cell system). Anat Sci Int. 2017;92(4):387-455. doi: 10.1007/s12565-017-0395-9	Review	Japan
55	2017	Denardin CC, Martins LA, Parisi MM, Vieira MQ, Terra SR, Barbé-Tuana FM, Borojevic R, Vizzotto M, Emanuelli T, Guma FC. Autophagy induced by purple pitanga (*Eugenia uniflora L.*) extract triggered a cooperative effect on inducing the hepatic stellate cell death. Cell Biol Toxicol. 2017;33(2):197-206. doi: 10.1007/s10565-016-9366-5	Original	Brazil
56	2017	Dias HB, Krause GC, Squizani ED, Lima KG, Schuster AD, Pedrazza L, Basso BS, Martha BA, de Mesquita FC, Nunes FB, Donadio MV, de Oliveira JR. Fructose-1,6-bisphosphate reverts iron-induced phenotype of hepatic stellate cells by chelating ferrous ions. Biometals 2017;30(4):549-558. doi: 10.1007/s10534-017-0025-y	Original	Brazil
57	2018	de Oliveira Mendes Ouriques FG, Caruso PB, da Silva GV, Dias H, Marques JR, Nunes FD, de Abreu Ferreira PM, de Oliveira JR. Antifibrotic effect of *pluchea sagitallis* (lam.) Cabrera aqueous extract in grx cell lineage. Int J Phytomedicine 2018;10(1):30-38. doi: 10.5138/09750185.1832	Original	Brazil
58	2018	Brea R, Motiño O, Francés D, García-Monzón C, Vargas J, Fernández-Velasco M, Boscá L, Casado M, Martín-Sanz P, Agra N. PGE2 induces apoptosis of hepatic stellate cells and attenuates liver fibrosis in mice by downregulating miR-23a-5p and miR-28a-5p. Biochim Biophys Acta Mol Basis Dis. 2018;1864(2):325-337. doi: 10.1016/j.bbadis.2017.11.001	Original	Spain
59	2018	Uschner FE, Schueller F, Nikolova I, Klein S, Schierwagen R, Magdaleno F, Gröschl S, Loosen S, Ritz T, Roderburg C, Vucur M, Kristiansen G, Lammers T, Luedde T, Trebicka J. The multikinase inhibitor regorafenib decreases angiogenesis and improves portal hypertension. Oncotarget 2018;9(90):36220-36237. doi: 10.18632/oncotarget.26333	Original	Germany/ Denmark/ Spain
60	2019	de Souza Basso B, de Mesquita FC, Dias HB, Krause GC, Scherer M, Santarem ER, de Oliveira JR. Therapeutic effect of Baccharis anomala DC. extracts on activated hepatic stellate cells. EXLI J 2019;18:91-105. doi: 10.17179/excli2018-1696	Original	Brazil
61	2019	Elias MB , Oliveira FL , Guma FCR , Martucci RB , Borojevic R , Teodoro AJ. Lycopene inhibits hepatic stellate cell activation and modulates cellular lipid storage and signaling. Food Funct. 2019;10(4):1974-1984. doi: 10.1039/c8fo02369g	Original	Brazil
62	2019	Ilha M, Moraes KDS, Rohden F, Martins LAM, Borojevic R, Lenz G, Barbé-Tuana F, Guma FCR. Exogenous	Original	Brazil

		expression of caveolin-1 is sufficient for hepatic stellate cell activation. J Cell Biochem. 2019;120(11):19031-19043. doi: 10.1002/jcb.29226		
63	2020	Azzam M, El Safy S, Abdelgelil SA, Weiskirchen R, Asimakopoulou A, de Lorenzi F, Lammers T, Mansour S, Tammam S. Targeting activated hepatic stellate cells using collagen-binding chitosan nanoparticles for siRNA delivery to fibrotic livers. Pharmaceutics 2020;12(6):590. doi: 10.3390/pharmaceutics12060590	Original	Egypt / Germany
64	2020	Winkler I, Bitter C, Winkler S, Weichenhan D, Thavamani A, Hengstler JG, Borkham-Kamphorst E, Kohlbacher O, Plass C, Geffers R, Weiskirchen R, Nordheim A. Identification of Pparγ-modulated miRNA hubs that target the fibrotic tumor microenvironment. Proc Natl Acad Sci U S A. 2020;117(1):454-463. doi: 10.1073/pnas.1909145117	Original	Germany
65	2020	Hübbers A, Hennings J, Lambertz D, Haas U, Trautwein C, Nevzorova YA, Sonntag R, Liedtke C. Pharmacological inhibition of cyclin-dependent kinases triggers anti-fibrotic effects in hepatic stellate cells in vitro. Int J Mol Sci. 2020;21(9):3267. doi: 10.3390/ijms21093267	Original	Germany
66	2021	Ribera J, Vilches C, Sanz V, de Miguel I, Portolés I, Córdoba-Jover B, Prat E, Nunes V, Jiménez W, Quidant R, Morales-Ruiz M. Treatment of hepatic fibrosis in mice based on targeted plasmonic hyperthermia. ACS Nano. 2021;15(4):7547-7562. doi: 10.1021/acsnano.1c00988	Original	Spain
67	2021	Awan T, Babendreyer A, Wozniak J, Alvi AM, Sterzer V, Cook L, Bartsch JW, Liedtke C, Yildiz D, Ludwig A. Expression of the Metalloproteinase ADAM8 is upregulated in liver inflammation models and enhances cytokine release *in vitro*. Mediators Inflamm. 2021;2021:6665028. doi: 10.1155/2021/6665028	Original	Germany
68	2021	de Oliveira CM, Martins LAM, de Sousa AC, Moraes KDS, Costa BP, Vieira MQ, Coelho BP, Borojevic R, de Oliveira JR, Guma FCR. Resveratrol increases the activation markers and changes the release of inflammatory cytokines of hepatic stellate cells. Mol Cell Biochem. 2021;476(2):649-661. doi: 10.1007/s11010-020-03933-1	Original	Brazil
69	2021	Arif E, Wang C, Swiderska-Syn MK, Solanki AK, Rahman B, Manka PP, Coombes JD, Canbay A, Papa S, Nihalani D, Aspichueta P, Lipschutz JH, Syn WK. Targeting myosin 1c inhibits murine hepatic fibrogenesis. Am J Physiol Gastrointest Liver Physiol. 2021;320(6):G1044-G1053. doi: 10.1152/ajpgi.00105.2021	Original	Spain/ USA
70	2021	Awan T, Babendreyer A, Wozniak J, Alvi AM, Sterzer V, Cook L, Bartsch JW, Liedtke C, Yildiz D, Ludwig A. Expression of the Metalloproteinase ADAM8 Is Upregulated in Liver Inflammation Models and Enhances Cytokine Release In Vitro. Mediators Inflamm. 2021;2021:6665028. doi: 10.1155/2021/6665028	Original	Germany
