# Supplementary material for: Genetic and Molecular Characterization of the Immortalized Murine Hepatic Stellate Cell Line GRX
Source: Cells. 2022 Apr 30;11(9):1504. doi: 10.3390/cells11091504 (PMC9102025; doi:10.3390/cells11091504)
Supplement: Supplementary file 1 [file cells-11-01504-s001.zip › Table S2.pdf]

Table S2. Antibodies used in Western blot analysis

Antibody	Cat.-no.	Company	Expected size (kDa)	Dilution	Clonality
α-SMA	CBL171-I	Sigma-Aldrich, Taufkirchen, Germany	45	1:1,000	m mAb
α-tubulin (B-7)	sc-5286	Santa Cruz Biotech., Santa Cruz, CA, USA	55	1:1,000	m mAb
β-actin	A5441	Sigma-Aldrich	43	1:10,000	m mAb
BST2	MBS9605302	MyBioSource, San Diego, CA, USA	22	1:1,000	p rAb
Caveolin-1	#3238	Cell Signaling Technology, Frankfurt am Main, Germany	21,24	1:1,000	r pAb
Col I	NB600-408	Novus Biologicals, Wiesbaden Nordenstadt, Germany	139	1:1,000	r mAb
Col III	22734-1-AP	Proteintech, Manchester, UK	139	1:1,000	r mAb
Col IV	ab6586	Abcam, Berlin, Germany	139	1:1,000	r pAb
Desmin (D93F5)	#5332	Cell Signaling	53	1:1,000	r mAb
E-Cadherin	AP20195PU-N	OriGene Technologies GmbH, Herford, Germany	130, 98	1:500	r pAb
Fibronectin	AB1954	Sigma-Aldrich	262	1:3,000	r pAb
GAPDH (6C5)	sc-32233	Santa Cruz Biotech.	37	1:1,000	m mAb
HNF-4α (C-19)	sc-6556	Santa Cruz Biotech.	54	1:750	p gAb
IL-6	AF-406-NA	R&D Systems, Wiesbaden, Germany	20-38	1:500	g pAb
Transferrin	17435-1-AP	Proteintech, Manchester, UK	77	1:1,000	r pAb
Vimentin	ab92547	Abcam	54	1:3,000	r mAb
MLV (p30)	ABIN956413	Antibodies-online GmbH, Aachen, Germany	30	1:1,000	m mAb
goat anti-rabbit IgG (H+L), HRP	#31460	Invitrogen, ThermoFisher Scientific, Schwerte, Germany	-	1:5,000	g
mouse anti-goat IgG (H+L), HRP	#31400	Invitrogen	-	1:5,000	m
goat anti-mouse IgG (H+L), HRP	#31430	Invitrogen	-	1:5,000	r

Abbreviations used are: g, goat; m, mouse; mAb, monoclonal antibody; pAb, polyclonal antibody; r, rabbit.
