# Supplementary material for: Genetic and Molecular Characterization of the Immortalized Murine Hepatic Stellate Cell Line GRX
Source: Cells. 2022 Apr 30;11(9):1504. doi: 10.3390/cells11091504 (PMC9102025; doi:10.3390/cells11091504)
Supplement: Supplementary file 1 [file cells-11-01504-s001.zip › Table S3.pdf]

Table S3. Overview of virus substrates used in this study

**HIV**	U5HIVB: ACTGCTAGAGATTTTCCACACTGACTAAA<br>5′ amine-U5HIVA: Am-TTTAGTCAGTGTGGAAAACTCTAGCAGT
**MLV**	U5MLVB: AATGAAAGACCCCCGCTGACGGGTAGTCAA<br>5′ amine U5MLVA: Am-TTGACTACCCGTCAGCGGGGGTCTTTCATT
**MMTV**	U5MMTVAB: AATGCCGCAGTCGGCCGACCTGAGGGTCACCG<br>5′ amine U5MMTVA: Am-CGGTGACCCTCAGGTCGGCCGACTGCGGCATT
